# Immunogenicity of a Lipopolysaccharide *Brucella melitensis* Vaccine in Goats: An Exploratory Study

**DOI:** 10.3390/vaccines13121209

**Published:** 2025-11-29

**Authors:** Alnakhli Naseer, Steven C. Olsen, Salman Mo, Joshua B. Daniels, Brian McCluskey

**Affiliations:** 1 Animal Population Health Institute, College of Veterinary Medicine and Biomedical Sciences, Colorado State University, Campus Stop 1644, Fort Collins, CO 80523, USA; mo.salman@colostate.edu (S.M.); bmccluskey@lonestar-epi.com (B.M.); 2 Infectious Bacterial Diseases Research Unit, National Animal Disease Center, Agricultural Research Service, U.S. Department of Agriculture, Ames, IA 50010, USA; steven.olsen@usda.gov; 3 Department of Microbiology, Immunology and Pathology, College of Veterinary Medicine and Biomedical Sciences, Colorado State University, Fort Collins, CO 80523, USA; josh.daniels@colostate.edu

**Keywords:** brucellosis, LPS vaccine, *Brucella melitensis*, humoral and cellular immunity, experimental challenge, goats

## Abstract

Background: *Brucella melitensis* is considered one of the most widespread zoonotic pathogens worldwide. Vaccination remains the most cost-effective strategy for controlling *B. melitensis* infection in small ruminants. Methods: In this study, we evaluated the immunologic responses and protection against experimental challenge in 18 goats vaccinated with either lipopolysaccharide (LPS) from *B. melitensis* strain 16M (LPS alone), LPS of *B. melitensis* strain 16M and MONTANIDE™ ISA 61 VG adjuvant (Seppic; 50 Bd national, 92250 La Garenne-Colombes, France) (LPS + ISA 61 VG), or saline as a control. Results: Goats (*n* = 6) vaccinated with LPS + ISA 61 VG had greater (*p* < 0.05) antibody responses than those that were nonvaccinated. Our data demonstrate that goats vaccinated with LPS + ISA 61 VG exhibited greater lymphocyte proliferative responses (*p* < 0.05) to the LPS antigen than those vaccinated with LPS alone at week 12 after vaccination. However, proliferative responses of peripheral blood mononuclear cell (PBMC) from goats vaccinated with LPS + ISA61VG did not differ (*p* > 0.05) from responses of PBMC from control goats. CD4+, CD8+, and γδ T cells from all vaccinated goats had negligible proliferation and failed to induce antigen-specific IFN-γ production. Control and vaccinated goats did not differ (*p* > 0.05) in their protection against abortion, uterine, fetal, mammary, or maternal infection. Conclusions: Our data suggests that LPS + ISA 61 VG induces a robust humoral response but negligible cellular responses. Our data also suggest that LPS + ISA 61 VG or LPS alone would not be efficacious for use as a vaccine in goats, but the LPS + ISA 61 VG inoculum may be beneficial as a booster. Additional trials would be necessary to evaluate the vaccine’s efficacy as a booster inoculation for small ruminants.

## 1. Introduction

Brucellosis is caused by virulent species of *Brucella* spp., specifically *B. abortus*, *B. melitensis*, and *B. suis*, which are the top worldwide bacterial zoonotic diseases, with more than 2.1 million new cases annually [1,2]. *B. melitensis* is the greatest cause of human brucellosis, primarily transmitted from its preferred host in small ruminants. In the absence of treatment, human brucellosis can be chronic and result in arthritis, abortion, cardiac issues, and sometimes mortality [3,4]. In addition to large economic costs associated with human disease, brucellosis causes significant losses in livestock by reproductive losses (abortion, weak fetuses, and infertility). Human brucellosis is primarily transmitted through contact with contaminated animal products, particularly non-pasteurized dairy products, or through direct exposure to infected animals. Addressing the disease in animal hosts, is the most efficient way to prevent human brucellosis, as people are essentially dead-end hosts [5]. Vaccination of animal reservoirs is the most cost-effective basis to control brucellosis [6], alongside serologic detection of antibodies and removal of infected animals. The *B. melitensis* strain Rev1 vaccine has been used to prevent brucellosis in small ruminants since the early 1950s [4,7]. The vaccine is efficacious but has limitations due to its tendency to cause abortions in pregnant animals, long-term seroconversion in vaccinates that cannot be differentiated from infection with field strains, shedding in milk, and high virulence in humans. Despite more than six decades of efforts, a more efficacious and safer *B. melitensis* vaccine for small ruminants than Rev1 has not been identified.

*B. melitensis* is classified as a Gram-negative, facultative intracellular bacterium in the Alphaproteobacteria class [8]. Its outer membrane is critical to the infection process, serving as the initial point of interaction between the bacterium and the host [9]. The major constituent, lipopolysaccharide (LPS), consists of three structural domains: (i) lipid A, which anchors the molecule within the outer membrane and contributes to its endotoxic activity; (ii) the inner and outer core oligosaccharides; and (iii) the O-antigen (O-polysaccharide, O-PS), a polymeric sugar chain extending outward from the cell surface. *B. melitensis* is typically found as a smooth LPS (S-LPS) strain that expresses the O-antigen on its LPS. Data suggest that *Brucella* LPS is essential for bacterial invasion, intracellular multiplication, and protecting the bacteria against complement-mediated lysis [10].

The primary innate defense mechanism against *B. melitensis* infection is initiated by the detection of its LPS by pattern recognition receptors (PRRs). These receptors identify specific molecular patterns known as pathogen-associated molecular patterns (PAMPs) [11,12]. When PRRs, such as Toll-like receptors, detect a particular PAMP, they initiate intracellular signaling in antigen-presenting cells, including macrophages and dendritic cells. This signaling cascade leads to an adaptive response that eventually targets infected cells and eliminates the intracellular pathogen [13]. Nevertheless, data indicates that *B. melitensis* infection may evade the innate immune response by diminished PAMP signaling in dendritic cells [13,14].

For protective immunity against brucellosis, it is widely acknowledged that antibody responses generated by vaccination do not correlate with long-term protection in natural hosts [13]. Effective immunity against Brucella infection requires the activation of CD8+ and CD4+ T lymphocytes. CD4+ T-cells are vital for coordinating the adaptive immune response and can differentiate into T helper (Th1) cells, producing key cytokines like interferon-γ (IFN-γ), interleukin-2 (IL-2), and tumor necrosis factor-α (TNF-α). They also support the growth and maintenance of CD8+ T-cells, which are essential for directly killing infected cells. Both CD4+ and CD8+ T-cells release cytokines that activate macrophages and dendritic cells, enhancing their ability to eliminate Brucella [13,15,16].

Combining the induction of antibodies against the Brucella O-polysaccharide (OPS) with a robust cell-mediated immune response may induce optimal immunity against brucellosis [17]. It has been suggested that this type of immune response may be achieved through the conjugation of Brucella OPS or by combining Brucella OPS with adjuvants or immunogenic Brucella proteins [18]. Current dogma is that both humoral and cellular immune responses are required for protective immunity against *B. melitensis* in small ruminants. In the current study, we tested this hypothesis using vaccines containing purified *B. melitensis* LPS alone or combined with an adjuvant. We hypothesized that *B. melitensis* LPS vaccines would induce robust humoral responses but anticipated less induction of cellular immune responses. Our study’s objective was to determine whether immune responses induced by two bioconjugate vaccines would induce humoral and cellular immune responses after vaccination and if they were efficacious in protecting pregnant goats against experimental challenge with virulent *B. melitensis*.

## 2. Materials and Methods

### 2.1. Bacterial Cultures

Smooth *B. melitensis* strain 16M was obtained from the culture collection at the National Animal Disease Center in Ames, IA. Frozen stock cultures were propagated on tryptose agar (Difco Laboratories, Detroit, MI, USA) supplemented with 5% bovine serum (TSA) for 72 h at 37 °C with 5% CO_2_. The bacteria were then inactivated as previously outlined [19]. Briefly, the cultures were washed with phosphate-buffered saline (PBS) and then suspended in methanol at a ratio of 1:2 (bacterial suspension:methanol). The suspension was incubated at 4 °C for 4 to 5 days. Inactivation was confirmed by plating on Kuzda and Morse (KM) plates. The inactivated cultures were used for subsequent extraction of lipopolysaccharide (LPS). All culture manipulations were conducted in a certified Biosafety Level 3 (BSL-3) cabinet.

### 2.2. Extraction and Quantification of B. melitensis LPS Using the Hot Phenol-Water Microextraction Method

LPS extraction was performed as previously described [20]. The procedure commenced with the collection of the culture through centrifugation, followed by resuspension in distilled deionized water (ddH_2_O). An equal volume of 90% phenol was subsequently added to the suspension, which was then shaken vigorously at a temperature range of 65–70 °C. The mixture was then centrifuged to facilitate the separation of the upper aqueous phase from the phenol phase. The aqueous layer was collected, and the phenol phase was re-extracted with ddH_2_O. To precipitate the LPS, 5–10 volumes of cold (−20 °C) 95% ethanol were added, followed by incubation at −20 °C overnight. The ethanol was then removed, and the resulting pellet was dissolved in 150 µL of ddH_2_O. This precipitation process was repeated once more to enhance LPS purity. The purified LPS was stored at −20 °C until further use.

LPS quantification was performed as previously described [21]. In brief, carbohydrate standards were prepared by diluting a 50:50 stock solution of 0.5 mg/mL sucrose and fructose to produce 1 mL aliquots with final concentrations of 0, 30, 60, 90, and 120 µg/mL. To conduct the assay, 200 µL of each carbohydrate standard, along with a control comprising 200 µL of 50 mM ethylenediaminetetraacetic acid (EDTA) and all sample solutions, were transferred into acid-washed glass test tubes. Both 200 µL of 5% phenol and 1 mL of 93% sulfuric acid were added to each tube. The reaction produced a yellow color, with the intensity of the color correlating to carbohydrate concentration. All reactions were measured using a spectrophotometer (OD490) (Synergy Neo2, BioTek, Santa Clara, CA, USA), and carbohydrate concentrations (LPS O-antigen) were calculated using a standard curve.

For the assessment of Brucella protein concentration within the inoculum, 1 µg of extracted LPS was treated with 2.5 µg of proteinase K and incubated at 59 °C overnight to digest any protein. The protein quantification within the LPS was carried out using Qubit^®^ Protein Assay Kits (ThermoFisher, New York, NY, USA, Cat. Q33211), in accordance with manufacturer guidelines.

For immunologic assays assessing antibody responses and mononuclear cell proliferation, extracted LPS was utilized as the antigen.

For the experimental challenge, *B. melitensis* strain 16M was cultured on tryptose agar for 72 h at 37 °C. Following incubation, the bacteria were collected from the agar surface using saline aspiration. Strain 16M suspensions were adjusted with a spectrophotometer to roughly 10^8^ colony-forming units (CFU) per mL, and concentrations of viable bacteria quantified through standard plate counts.

### 2.3. Emulsification of LPS and Montanide ISA 61 VG

A vaccine formulation containing lipopolysaccharide (LPS) was prepared using ISA 61 VG (SEPPIC) as the adjuvant. Purified Brucella LPS (1.4 mL, 730 µg) was combined with 2.1 mL of ISA 61 VG to achieve a 40:60 (*v*/*v*) antigen-to-adjuvant ratio, following the manufacturer’s instructions. Emulsification was performed using a two-way syringe system, consisting of 20 low-speed cycles (8 s each) followed by 60 high-speed cycles (1 s each). The emulsion was considered stable when droplets floated on the surface and retained their water-in-oil consistency.

### 2.4. Animals and Inoculation

All studies using animals were performed with approval and oversight from the Institutional Animal Care and Use Committee (IACUC) at the National Animal Disease Center. Eighteen adult female goats were obtained from a brucellosis-free herd. Following a two-week acclimatization period, the goats were randomly assigned to three treatments (*n* = 6/trt). The three treatments were saline (control), 400 µg of LPS alone, and 292 µg of LPS with MONTANIDE™ ISA 61 VG adjuvant. All inoculums were administered subcutaneously in the cervical region. Continuous monitoring was conducted to observe any potential adverse clinical signs in the goats, such as fever, swelling at the injection site or regional lymph nodes, anorexia, lameness, abscess formation at the injection site, and diarrhea.

### 2.5. Post-Vaccination Serologic Responses

Blood samples were obtained by jugular venipuncture and placed into serum separator tubes. After centrifugation, the serum was divided into aliquots and stored at −80 °C until analysis. To evaluate the humoral immune response to LPS, an enzyme-linked immunosorbent assay (ELISA) was conducted to quantify total LPS-specific immunoglobulin G (IgG), following previously described methods [22]. In brief, 96-well flat-bottom plates were coated overnight at 4 °C with 1 µg/well of LPS antigen prepared in carbonate–bicarbonate buffer (0.18 M carbonate, 0.028 M sodium bicarbonate, pH 9.6). Plates were then blocked twice for 15 min with SuperBlock™ (Thermo Fisher Scientific, Waltham, MA, USA). Serum samples diluted 1:800, 1:1600, and 1:3200 were added in triplicate and incubated for 2 h at room temperature (RT). After four washes with phosphate-buffered saline containing 0.05% Tween 20 (PBST), 100 µL of peroxidase-conjugated AffiniPure Rabbit Anti-Goat IgG (H + L) (Jackson ImmunoResearch Laboratories, Inc., West Grove, PA, USA) diluted 1:5000 was added. Following a 1 h incubation at RT, the plates were washed four times and developed with a TMB substrate kit (Thermo Scientific, Waltham, MA, USA) as per the manufacturer’s guidelines. The reaction was terminated by adding 100 µL of 0.18 M sulfuric acid, and optical density (OD) was measured at 450 nm using a microplate reader (Synergy Neo2, BioTek, Santa Clara, CA, USA).

### 2.6. Peripheral Blood Mononuclear Cells and Proliferative Responses

At 12 weeks post-vaccination, jugular blood samples were collected into acid-citrate dextrose tubes, and PBMCs were isolated by density gradient centrifugation [16]. Cell viability was assessed using the trypan blue exclusion method. PBMCs were resuspended in complete RPMI 1640 medium (cRPMI; Gibco Life Technologies, Thermo Fisher Scientific, Waltham, MA, USA) at a final concentration of 1 × 10^7^ cells/mL. Aliquots of 50 µL (5 × 10^5^ cells) were dispensed in duplicate into microtiter plate wells. Each well was filled with either 100 µL of cRPMI medium alone or medium containing LPS at varying concentrations (ranging from 2 µg to 0.125 µg LPS per well, using a 1:2 dilution series). Concanavalin A (ConA; 0.5 µg/well; Sigma, St. Louis, MO, USA) served as a positive control. The plates were incubated for 7 days at 37 °C in a 5% CO_2_ atmosphere, after which cells were pulsed for 18 h with 1.0 µCi of [^3^H]-thymidine per well. Radioactive incorporation was quantified following cell harvesting onto glass fiber filters using a liquid scintillation counter (PerkinElmer, Hopkinton, MA, USA) [22].

Proliferative responses of PBMC were also assessed by flow cytometry at week 15, as previously described [16]. PBMCs were stained using a 1:10 dilution of CellTrace Violet (eBioscience, Waltham, MA, USA, Thermo Fisher Scientific) according to the manufacturer’s instructions and resuspended in complete RPMI media. PBMCs were then plated at a concentration of 1 × 10^6^ cells per well in 96-well flat-bottom plates. Wells contained unstimulated (RPMI only), 0.1 µg/well of LPS antigen, ConA, and irradiated *B. melitensis* 16M bacteria (1 × 10^7^ CFU). After incubation for 7 days at 37 °C in a 5% CO_2_, cells were treated with either 1× GolgiStop solution (eBioscience, Thermo Fisher Scientific) alone, or Cell Stimulation Cocktail for approximately 16 h prior to harvesting as per manufacturer recommendations. Cells were subsequently processed for flow cytometry staining as previously described methods [16]. After transferring to round-bottom 96-well plates, centrifugation, and 2 washes in Dulbecco’s phosphate-buffered saline (DPBS), cells were incubated with a fixable viability dye (Invitrogen EBioscience Fixable Viability Dye eFlour 450, Fisher Scientific) and stained for surface markers. Cell surface markers included: CD4 (clone S-17D, from Washington State University, and an anti-mouse antibody from BD Bioscience, Franklin Lakes, NJ, USA), CD8 (clone St8, from Washington State University, and FITC anti-mouse from BioLegend, San Diego, CA, USA), and γδ T-cells (which recognize the TCR1-N24 δ chain, sourced from Washington State University, along with an anti-mouse antibody, Clone R12-3 (RUO), from BD Bioscience). Cells were fixed and permeabilized using the BD Cytofix/Cytoperm™ kit (BD Bioscience) in accordance with the manufacturer’s instructions and then stained with a PE-conjugated anti-bovine IFN-γ antibody (Bio-Rad, Hercules, CA, USA). The PBMCs were rinsed twice with wash buffer (BD Biosciences) and once with FACS buffer, then finally resuspended in FACS buffer for analysis. Flow cytometry data were collected on a BD FACSymphony™ A5 flow cytometer (BD Bioscience) equipped with DIVA software(v9.3). Data were analyzed using FlowJo^®^ (FlowJo v10.10).

### 2.7. B. melitensis Experimental Challenge

Goats were naturally bred under field conditions, and pregnancy was confirmed using a serological assay that detects pregnancy-specific protein B (Bovine Pregnancy Test, Rapid Visual Kit; IDEXX, Westbrook, ME, USA). At approximately two and a half months of gestation, pregnant goats were transferred to the Agricultural Biosafety Level 3 (AgBSL-3) facility at the National Animal Disease Center (NADC), Ames, Iowa, where they were housed individually for the remainder of the experiment. Each goat was challenged intraconjunctivally with *B. melitensis* strain 16M, receiving 50 µL of inoculum per eye containing approximately 10^7^ CFU [23,24]. The concentration of viable bacteria in the inoculum was verified by standard plate count methods. A successful experimental challenge was confirmed by isolating the challenge strain from conjunctival swabs collected five days post-inoculation [25]. The recovered isolates were identified as *Brucella* by means of polymerase chain reaction (PCR) using Brucella-specific primers targeting the omp2a gene [26].

### 2.8. Post-Challenge Serologic Responses

Blood samples were collected through jugular venipuncture prior to and at two weeks following experimental challenge. Additional blood samples were obtained at necropsy following parturition or abortion. Serum was obtained by centrifugation, sterilized through filtration, aliquoted into 1 mL portions, and stored at −70 °C. Post-challenge serologic titers against *B. melitensis* were measured using the ELISA method described above.

### 2.9. Necropsy and Bacterial Culture Procedures

Within 48 h after parturition, or immediately following abortion, goats were euthanized through intravenous injection of sodium pentobarbital. Maternal samples collected during necropsy included milk from two quarters, blood, and various lymph nodes (prescapular, supra-mammary, internal iliac, hepatic, retropharyngeal, mandibular, bronchial, and parotid). Additional samples included the liver, placentome or caruncle, vaginal swab, spleen, mammary gland tissue from two quarters, and lung. Fetal samples obtained included the gastric contents, lung, liver, blood, bronchial lymph node, rectal swabs, and spleen. Swabs and fluid samples were inoculated directly onto KM media. Tissue samples were weighed, homogenized in 0.15 M NaCl using a tissue grinder, and then plated onto a KM medium. For tissues with high bacterial numbers, homogenates were serially diluted in saline. The level of colonization (colony-forming units (CFU)/g) was determined by standard plate counts. Samples were incubated at 37 °C in a 5% CO_2_ atmosphere. *B. melitensis* was identified by colony morphology, growth characteristics, and a Brucella-specific real-time PCR (RT-PCR) assay targeting the outer membrane protein 2 (Table 1). Tissue suspensions were also tested using the Brucella-specific RT-PCR assay.

### 2.10. Definitions

Abortion was defined as the premature expulsion of a nonviable fetus infected with *Brucella* after experimental challenge. The dam or fetus was considered infected if at least one colony of *B. melitensis* was recovered from any tissue collected during necropsy. Mammary infection was confirmed by isolating the 16M challenge strain from mammary gland tissue, milk, or supramammary lymph nodes. Uterine infection was identified by recovery of the 16M strain from internal iliac lymph nodes, vaginal swabs, or placentomes. Fetal infection was defined by the isolation of the 16M strain from any fetal tissue sample.

### 2.11. Statistical Analysis

Responses from all experimental goats, measured by antibody titer, flow cytometry, [^3^H]-thymidine incorporation, and tissue colonization, before and after challenge, were considered the primary outcomes for comparison. The clinical outcome, indicated by the occurrence of abortion and 16M infection, was considered the secondary outcome.

Data of the primary outcomes were summarized statistically using means (for normally distributed data) or medians (for non-normally distributed data), along with measures of dispersion (Standard error or range), as appropriate for the tests performed [27].

A one-way ANOVA [28] was used for flow cytometry, [^3^H]-thymidine, and tissue colonization, and a two-way repeated-measure ANOVA [28] was used for antibody titers, with treatment group and time as the study variables. Prior to applying ANOVA, a normality test (Shapiro–Wilk) [28,29] was performed to confirm the normal distribution of each outcome. If an outcome failed the normality test, the Kruskal–Wallis or Friedman non-parametric test was applied instead.

Chi-square analysis was used to assess the association between experimental groups and the occurrence of abortion and 16M infection. Statistical significance was set at *p* < 0.05. All statistical tests were conducted using SAS v9.4M9 statistical software (SAS Institute, Inc., Cary, NC, USA).

Due to the exploratory nature of this experimental study and the small sample size, statistical testing of the generated data was not intended for population-level inference. Instead, the analyses aimed to compare group differences and to inform sample size estimation for future large-scale field experimental or observational studies.

## 3. Results

The normality test was conducted prior to the analysis. Proliferation, flow cytometry, and colonization data met the normality requirement and were analyzed using one-way ANOVA. However, the serologic data did not meet the normality requirement, and therefore, the Friedman non-parametric test was applied.

### 3.1. LPS Extraction and Quantification

LPS was successfully extracted from *B. melitensis* strain 16M. The extracted LPS product contained 34.7 µg/mL of protein and the concentration of the LPS in our stock was determined to be 280 µg/mL. The average vaccination dosages for treatments two and three were determined to be 400 µg and 292 µg LPS, respectively.

### 3.2. Clinical Signs

In the vaccinated group, two goats were excluded from the experimental challenge, including one diagnosed with lameness and another goat that underwent parturition before challenge. It could not be conclusively determined whether the observed lameness was associated with the vaccination or occurred incidentally, as no other clinical abnormalities were noted. Apart from this isolated case, no local (e.g., injection-site swelling) or systemic adverse events were observed in the remaining vaccinated goats following vaccine administration. 

### 3.3. Post-Vaccination Serologic Responses

**Figure 1:**
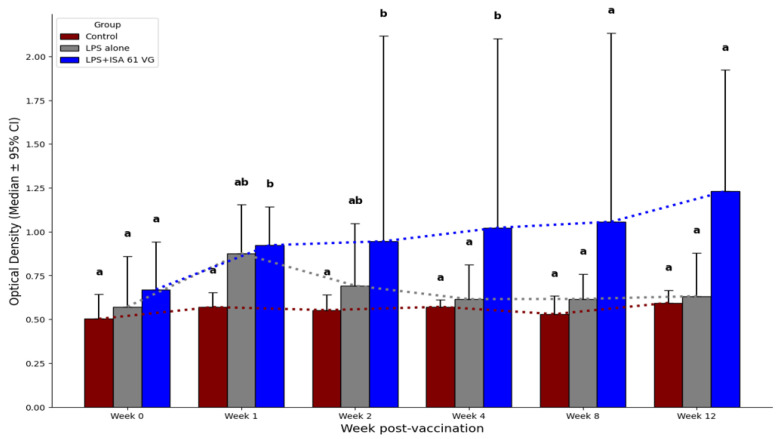
Median IgG serologic responses to LPS antigens of non-vaccinated goats (*n* = 6) and goats vaccinated with either LPS alone (*n* = 6) or LPS + ISA61VG (*n* = 6). Data are presented as a median optical density (OD) ± 95% confidence interval for serum samples diluted 1:1600. Medians bearing different letters (a and b) indicate significant differences (*p* < 0.05) between treatment groups at the same time point.

When compared to non-vaccinated goats, those vaccinated with LPS + ISA61VG demonstrated greater (*p* < 0.05) median IgG responses to the LPS antigen at 1, 2, and 4 weeks. Goats vaccinated with LPS + ISA61VG had greater median titers (*p* < 0.05) only at 4 weeks after vaccination when compared to LPS alone. When compared to non-vaccinated goats, goats vaccinated with LPS alone demonstrated a trend for greater median antibody responses to the LPS antigen at week 1 (*p* = 0.06). Goats vaccinated with LPS + ISA61VG- tended (*p* = 0.06) to have greater median antibody responses at 8 and 12 weeks when compared to the other two treatments (Figure 1). Our data suggest that LPS + ISA61VG-vaccinated goats displayed more robust humoral responses against Brucella LPS than those receiving the other two treatments.

### 3.4. Post-Vaccination Lymphocyte Proliferation

**Figure 2:**
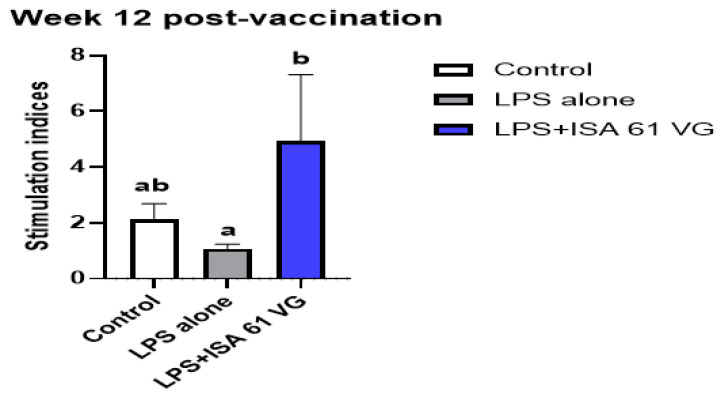
Mean proliferative responses to 0.1 µg LPS by PBMC from non-vaccinated goats (*n* = 6) or goats vaccinated with LPS alone (*n* = 6) or LPS + ISA61VG (*n* = 6). Cells were incubated at 37 °C in 5% CO_2_ for 7 days and then pulsed with [^3^H]-thymidine for 18 h. Data are presented as mean stimulation indices ± SEM, and means labeled with different lowercase letters (a and b) indicate significant differences (*p* < 0.05) comparing treatment groups at the same time point.

Goats vaccinated with LPS + ISA61VG had greater mean proliferative responses (*p* < 0.05) to LPS antigens at week 12 after vaccination when compared to responses of goats inoculated with LPS alone. The mean proliferative responses of goats immunized with LPS + ISA61VG did not differ (*p* = 0.10) at this sampling time from mean responses of non-vaccinated goats (Figure 2). These data suggest some antigen-specific proliferative responses were present after LPS + ISA61VG vaccination.

### 3.5. Post-Vaccination T Cell Population Subsets, Proliferation, and IFN-γ Responses

**Figure 3:**
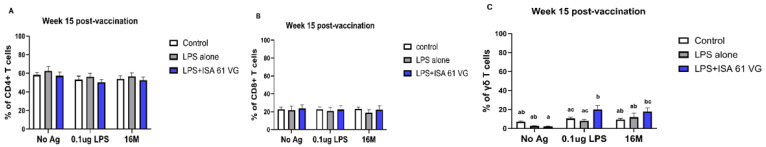
Frequency of CD4+ (**A**), CD8+ (**B**), and γδ (**C**) T cell subsets in PBMC from control (white, *n* = 6), LPS + ISA61VG- (blue, *n* = 5), and LPS-vaccinated (gray, *n* = 5) goats after in vitro incubation for 7 days without stimulation (No Ag) and when incubated with 0.1 μg LPS or killed 16M bacteria. Data are presented as the percentage of CD4+ (**A**), CD8+ (**B**), and γδ (**C**) T cells within the gated cell populations. Means labeled with different lowercase letters (a, b, and c) indicate significant differences (*p* < 0.05).

Flow cytometric analysis indicated that the frequencies of CD4+ and CD8+ T cells did not differ (gating scheme for the analysis shown in Supplementary Figure S1) between vaccinated goats at week 15 (Figure 3A–C). However, goats vaccinated with LPS + ISA61VG demonstrated increased frequencies of γδ T cells when stimulated with Brucella LPS compared to those vaccinated with LPS alone or the control group. Furthermore, goats that received the LPS + ISA61VG exhibited notably higher frequencies of γδ T cells after stimulation with either LPS or killed 16M bacteria when compared to their unstimulated cells (Figure 3C). Overall, the frequencies of CD4+ and CD8+ T cell subsets were stable, with CD4 T cells comprising most of the circulating pool of T cells (Figure 3A).

**Figure 4:**
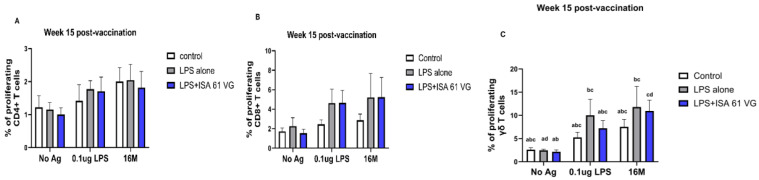
Proliferative responses of CD4+ (**A**), CD8+ (**B**) and γδ T (**C**) cells within PBMC isolated from control (white, *n* = 6), LPS + ISA61VG- (blue, *n* = 5), and LPS-vaccinated (gray, *n* = 5) goats at 15 weeks after vaccination, after in vitro incubation for 7 days without stimulation (No Ag) and when incubated with 0.1 μg LPS or killed 16M bacteria. Cells were analyzed for proliferation using a membrane-based dye. Data are shown as mean frequencies ± SEM, and different lowercase letters (a, b, c, and d) indicate significant differences (*p* < 0.05).

**Figure 5:**
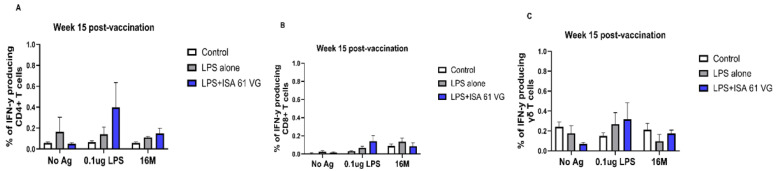
IFN-γ production of CD4+ (**A**), CD8+ (**B**) and γδ T cells (**C**) populations of PBMC from PBMC isolated control (white, *n* = 6), LPS + ISA61VG- (blue, *n* = 5), and LPS-vaccinated (gray *n* = 5) goats at 15 weeks after vaccination, after in vitro incubation for 7 days without stimulation (No Ag) and when incubated with 0.1 μg LPS or killed 16M bacteria. Data are presented as mean frequencies of cells expressing IFN-γ ± SEM.

We also compared frequencies of PBMCs proliferating and producing IFN-γ after stimulation with 0.1 μg LPS, killed 16 M bacteria, or when unstimulated. Our data suggest that CD4+, CD8+, and γδ T cells from vaccinated goats had negligible proliferation and failed to produce IFN-γ (Figure 4 and Figure 5), and responses did not differ (*p* > 0.05) from those of the control treatment. There was a nonsignificant trend for CD8 T cells from LPS alone or LPS + ISA61VG vaccinates to demonstrate increased proliferation after stimulation with Brucella LPS or killed 16M bacteria (Figure 4B). Goats in both vaccination treatments had greater (*p* < 0.05) frequencies of proliferating γδ T cells after stimulation with Brucella LPS or killed 16M bacteria when compared to unstimulated cells (Figure 4C). Goats in both vaccination treatments showed no significant differences (*p* > 0.05) in IFN-γ-producing CD4+, CD8+, and γδ T cells in response to Brucella LPS or killed 16M bacteria compared to unstimulated cells (Figure 5A–C). Our data suggest that most of the cells responsive to Brucella antigens after vaccination were γδ T cells, and CD4+ and CD8+ T cells do not proliferate or produce IFN-γ in response to the vaccination treatments evaluated.

### 3.6. Post-Challenge Results

**Figure 6:**
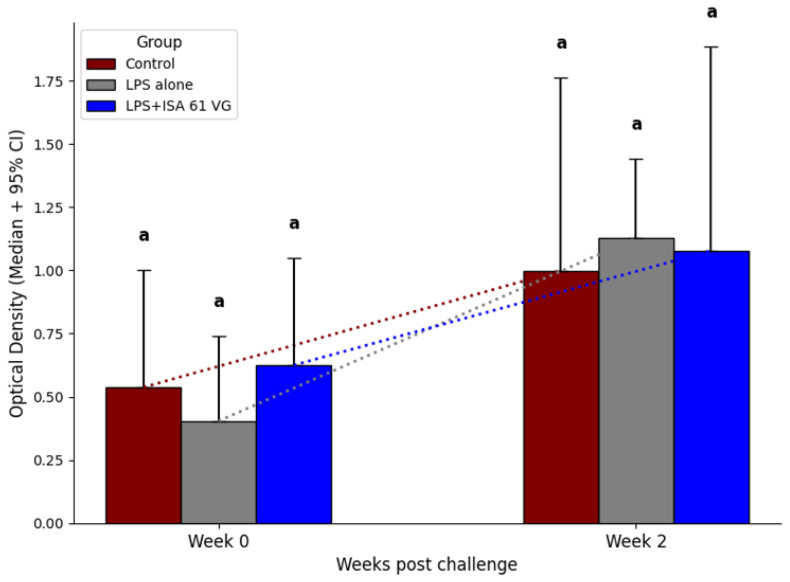
Serologic IgG responses to killed 16M bacteria in an ELISA assay prior to experimental challenge and at 2 weeks after challenge in an ELISA in nonvaccinated goats (burgundy, *n* = 6) and LPS alone (gray, *n* = 5) or LPS + ISA61VG vaccinated goats (blue, *n* = 5). Serum samples were collected at pre- and post-challenge. Data is from samples diluted 1:1600 and presented as median optical density ± 95%CI. Medians with different superscripts (a and b) are different (*p* < 0.05).

Before the challenge and at 2 weeks post challenge, median ELISA titers to killed 16M bacteria did not differ (*p* > 0.05) between the two vaccination treatments and the control group (Figure 6).

All treatments exhibited high rates of abortion and colonization in uterine, mammary, fetal, and maternal tissues (Table 2). In most instances, fetuses were severely autolytic with significant amounts of bacterial contamination, which prevented numeration of Brucella colonization. Control and vaccination treatments did not differ (*p* > 0.05) in the frequency of Brucella colonization in tissues (Table 3) and in milk, blood, or other samples. When evaluated across tissues, the placentome, fetal lung, and fetal liver had the highest levels of Brucella colonization (Table 3).

## 4. Discussion

The results of this study indicate that vaccination with LPS alone or combined with ISA 61 VG adjuvant is not efficacious in protecting goats against experimental challenge with virulent *B. melitensis* 16M strain. We attribute this lack of efficacy to a failure to induce cellular immunity, as we failed to find differences between treatments in antigen-specific responses in T cell subsets. As with many facultative intracellular pathogens, cell-mediated immunity is considered crucial in providing long-term protection against Brucella [30]. The OPS of lipopolysaccharide serves as the major immunodominant antigen of *B. melitensis*, and nearly all serological tests for brucellosis are based on measuring antibody responses to this antigen [31]. Since our study utilized non-living, subunit vaccines, the observed lack of cell-mediated responses could be related to how these vaccines are processed within antigen-presenting cells and presented to T cell populations.

Our data demonstrate that the LPS + ISA 61 VG vaccine induced stronger humoral responses when compared to the other treatments, but cellular immune responses did not differ from other treatments. It is hypothesized that vaccine strains derived externally and entering the antigen-processing cell through phagocytosis are presented via the Class II MHC (major histocompatibility complex) or exogenous pathway. In contrast, vaccine strains synthesized within the antigen-presenting cell’s cytoplasm and transported to the endoplasmic reticulum can be presented through Class I MHC and processed via the endogenous pathway. The intracellular location and/or method of entry of vaccine strains into the cell appears to be critical, as processing via the endogenous pathway tends to evoke a Th1 response associated with cell-mediated immunity. Vaccine strains (such as LPS alone or LPS plus ISA 61 VG in the current study) processed through the exogenous pathway are usually associated with humoral Th2 responses, which do not protect against intracellular pathogens. This may explain why current brucellosis vaccines are almost exclusively composed of live bacteria, whereas vaccinations with killed bacteria typically fail to provide adequate efficacy against the pathological effects of Brucella [32,33].

Cell-mediated immunity plays a vital role in providing protection against brucellosis; however, humoral responses could also contribute to vaccine efficacy. Previous studies have identified humoral mechanisms contributing to protection against *B. abortus* infection, including antibodies targeting Brucella LPS, activation of complement-mediated killing, antibody-dependent cytotoxicity, and enhanced phagocytosis by opsonization [34]. Others have hypothesized that anti-Brucella OPS antibodies, generated either by vaccination or natural infection, may help to prevent infection by activating complement-mediated bacterial killing mechanisms [35]. Notably, strong antibody responses have been documented in elk vaccinated with the RB51 strain, which have a prolonged bacteremia and lower PBMC proliferative responses after vaccination as compared to responses observed in cattle and bison [36]. Elk also exhibit fewer pathological effects (i.e., abortions) after experimental challenge. The lack of efficacy from vaccines evaluated in the current study suggests that combining the induction of anti-*Brucella* OPS humoral responses using a subunit vaccine with a robust cell-mediated immune response from a live vaccine might offer greater efficacy against brucellosis. Further research is warranted to characterize the efficacy of this combined approach.

Abortion rates were high among all vaccinated goats, which also exhibit significant colonization (CFU/gm) in targeted tissues. Some tissues selected for evaluation were chosen because they serve specific roles: the parotid lymph node represents lymphatic tissues at the site of experimental challenge, the placentome is localized in reproductive tissues where pathologic effects occur, the supramammary lymph node correlates with infection in the mammary gland where shedding may occur in milk, and the prescapular lymph node represents a lymph node not typically associated with Brucella localization but which may be colonized when wide spread in vivo infection occurs. Abortion is the most significant route for brucellosis transmission among ruminants. Higher bacterial counts in the uterine environment are associated with an increased risk of lateral transmission of brucellosis through expelled fluids, placental tissues, or fetal tissues [37]. Therefore, the combination of increased abortions and high reproductive colonization would not reduce disease transmission in our vaccinated goats if similar infections occurred under field conditions.

A glycoconjugate (GC) vaccine, composed of LPS and outer membrane protein (OMP) derived from *B. abortus* S19, has been demonstrated to elicit robust cell-mediated immune responses in both mice and bovine calves [38,39]. Additionally, Mukherjee reported that a 100 µg dose of the S19GC vaccine administered subcutaneously to adult female cattle can provide both therapeutic and prophylactic effects [40]. Similarly, in 1991, Jacques et al. demonstrated the effectiveness of a Brucella O-polysaccharide (PS)-bovine serum albumin (BSA) conjugate vaccine in protecting mice against *B. melitensis* H38 [41]. Our data in goats differ from mice in that vaccination with LPS alone or in combination with ISA 61 VG exhibited less protection against experimental challenge with *B. melitensis* 16M strain.

The current study highlights the critical need to assess potential Brucella vaccine strains in specific targeted species. Regrettably, most candidate vaccines for *B. melitensis* have only been tested in murine models, where disease pathogenesis may significantly differ from that in ruminant hosts. Unlike outbred domestic livestock, murine models are inbred, which can influence observed immune responses. For example, ruminants typically have a higher proportion of circulating T cells expressing γδ markers. Furthermore, while infection in mice is commonly evaluated through colonization in the liver and spleen, in ruminant hosts, the infection primarily localizes within lymphatic tissues [42,43,44].

Our data suggest that a single vaccination with LPS + ISA 61 VG or LPS alone does not offer sufficient efficacy in goats. However, the strong humoral responses elicited by LPS + ISA 61 VG could be beneficial for use as a booster vaccine. Although humoral responses (IgG levels) increased, we did not assess the functional properties of these antibodies, including their affinity, subclass distribution, or mucosal IgA responses. It is important to note that our study is limited by the small number of goats involved and may not reflect anamnestic immune responses in goats that were previously vaccinated or exposed to brucellosis. The current study is also limited by not including the Rev 1 vaccine, which is essential for a favorable control comparison. Furthermore, one limitation of the present study is that the exact antigen specificity of the humoral response remains undetermined. Typically, the effectiveness of brucellosis vaccines in field conditions exceeds that observed in experimental settings, where all animals are pregnant and exposed to a virulent Brucella strain during mid-gestation, the period of highest susceptibility to brucellosis. Until a more effective vaccine is developed, the Rev1 vaccine remains the preferred vaccine to reduce brucellosis in regions where it is endemic in goats.

## 5. Conclusions

In the present study, we evaluated immune responses and protection against experimental challenge following the vaccination of goats with LPS + ISA 61 VG, LPS alone, or saline as a control. Goats vaccinated with LPS + ISA 61 VG showed significantly higher antibody responses compared to those in the control group. Additionally, antigen-specific proliferative responses were observed in goats vaccinated with LPS + ISA 61 VG compared with those receiving LPS alone or the control. However, CD4+, CD8+, and γδ T cells from vaccinated goats showed minimal proliferation and did not produce IFN-γ. No significant differences were detected in the protection against abortion or in fetal, uterine, mammary, or maternal infections between vaccinated and control groups. Taken together, our findings indicate that a single vaccination with LPS + ISA 61 VG or LPS alone does not confer sufficient protection in goats. Nonetheless, the humoral responses induced by LPS + ISA 61 VG suggest potential value as a booster vaccine. Further studies are warranted to evaluate its efficacy as a booster vaccination in goats.

## Figures and Tables

**Table 1:** Primers and probes against the *Brucella* outer membrane protein 2 used in real-time polymerase chain reaction assay.

Forward primer	CCCAAGCATTGTCTTCAGCAACAG
Reverse primer	TGG TCT GAA GTA TCA GGC TAC GCA
Probe	56-FAM/ACCTTGGTGTAGGAAACTTCCGGCGT/31ABkFQ
Cycling 30 s 60 °C, 2 min 95 °C, and 40 cycles of 95 °C for 20 s and 60 °C for 1 min	

**Table 2:** Efficacies of LPS alone or combined with ISA 61 VG vaccination in protecting against experimental challenge at midgestation with 10^7^ *B. melitensis* strain 16M. Rate of abortion or infection (% [no. aborted or infected/total]).

Vaccination Group	N	Abortion	Uterine Infection *	Mammary Infection **	Fetal Infection ***	Remaining Maternal Tissues ****
control	6	100 (6/6)	100 (6/6)	100 (6/6)	80 (5/6)	100 (6/6)
LPS alone	5	80 (4/5)	60 (3/5)	100 (5/5)	60 (3/5)	100 (5/5)
LPS + ISA61VG	5	80 (4/5)	80 (4/5)	100 (5/5)	80 (4/5)	100 (5/5)

* Internal iliac lymph node, vaginal swab, and/or placentome; ** supramammary lymph node, milk, and/or mammary gland tissues; *** fetal gastric contents, liver, spleen, lung, rectal swab or bronchial lymph node; **** all maternal tissues not included in uterine infection or mammary infection column.

**Table 3:** Tissue colonization at parturition after conjunctival infection of control or vaccinated goats with *B. melitensis* strain 16M.

	Culture Positive/Total	Mean CFU/gm ± SEM ^†^
Maternal		
Lung	13/16 (81%) ^ab^	2.23 ± 0.44 ^a^ (*n* = 6)
Liver	13/16 (81%) ^ab^	2.03 ± 0.22 ^a^ (*n* = 7)
Spleen	14/16 (87%) ^ab^	2.45 ± 0.44 ^a^ (*n* = 6)
Bronchial LN	15/16 (93%) ^ab^	3.06 ± 0.22 ^a^ (*n* = 6)
Hepatic LN	16/16 (100%) ^ab^	2.83 ± 0.23 ^a^ (*n* = 11)
Iliac LN	13/16 (81%) ^ab^	2.90 ± 0.34 ^a^ (*n* = 7)
Mandibular LN	16/16 (100%) ^ab^	2.64 ± 0.15 ^a^ (*n* = 11)
Mesenteric LN	12/16 (75%) ^ab^	1.85 ± 0.30 ^a^ (*n* = 7)
Parotid LN ^‡^	30/32 (93%) ^a^	3.18 ± 0.29 ^a^ (*n* = 12)
Prescapular LN	14/16 (87%) ^ab^	2.24 ± 0.26 ^a^ (*n* = 6)
Retropharyngeal LN ^‡^	29/32 (90%) ^a^	2.71 ± 0.18 ^a^ (*n* = 12)
Supramammary LN ^‡^	32/32 (100%) ^a^	2.58 ± 0.20 ^a^ (*n* = 14)
Mammary gland ^‡^	28/32 (87%) ^a^	2.35 ± 0.26 ^a^ (*n* = 12)
Placentome	15/16 (93%) ^ab^	10.33 ± 0.23 ^c^ (*n* = 10)
Fetal		
Fetal Lung	7/16 (43%) ^b^	6.25 ± 2.42 ^b^ (*n* = 3)
Fetal liver	10/16 (62%) ^b^	7.13 ± 2.19 ^b^ (*n* = 3)
Fetal Spleen	7/16 (43%) ^b^	3.93 ± 0.86 ^a^ (*n* = 2)

^†^ Mean colony-forming units per gram in tissues PCR positive for Brucella and for which individual colonies could be numerated. ^‡^ Samples evaluated from both right and left sides of the tissue. Means bearing different superscripts are significantly different (*p* < 0.05).

## Data Availability

The raw data supporting the conclusions of this article will be available upon request from the corresponding author. The data are not publicly available due to privacy restrictions.

## Supplementary Materials

The following supporting information can be downloaded at: https://www.mdpi.com/article/10.3390/vaccines13121209/s1, Figure S1: Gating strategy for flow cytometry analysis.
