# Supplementary material for: Immunogenicity of a Lipopolysaccharide Brucella melitensis Vaccine in Goats: An Exploratory Study
Source: Vaccines (Basel). 2025 Nov 29;13(12):1209. doi: 10.3390/vaccines13121209 (PMC12737732; doi:10.3390/vaccines13121209)
Supplement: Supplementary file 1 [file vaccines-13-01209-s001.zip › vaccines-3970619-supplementary.pdf]

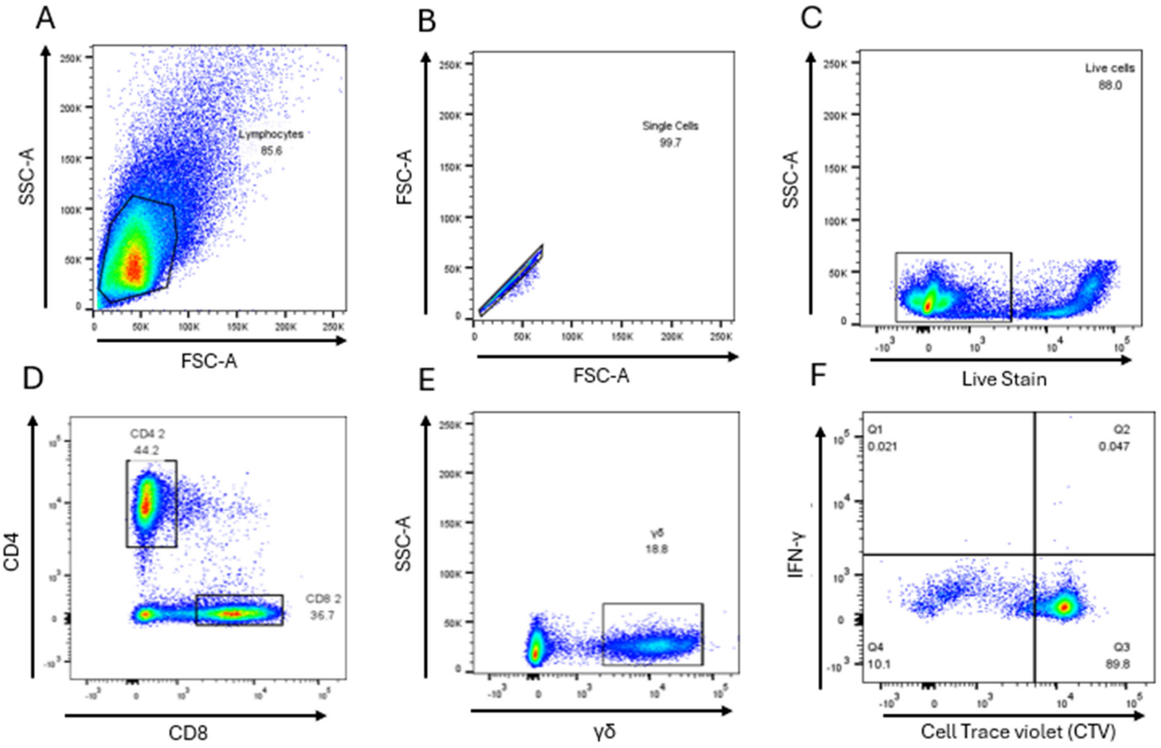

Supplementary Figure S1. Gating strategy for flow cytometry analysis. Shown are representative dot plots for lymphocyte gating via forward scatter (FSC-A) vs. side scatter (SSC-A) (A), singlet discrimination (B), gating on live events(C). Live cells were then further gated for CD4, CD8 and γδ expressions (D and E) and CD4, CD8 and γδ T cells were evaluated for IFN-γ vs. Cell Trace violet (CTV), indicative of proliferation.
